# Myelin Basic Protein Induces Neuron-Specific Toxicity by Directly Damaging the Neuronal Plasma Membrane

**DOI:** 10.1371/journal.pone.0108646

**Published:** 2014-09-25

**Authors:** Jie Zhang, Xin Sun, Sixin Zheng, Xiao Liu, Jinghua Jin, Yi Ren, Jianhong Luo

**Affiliations:** 1 Department of Neurobiology, Key Laboratory of Medical Neurobiology of the Ministry of Health of China, Zhejiang Province Key Laboratory of Neurobiology, Zhejiang University School of Medicine, Hangzhou, Zhejiang, China; 2 Division of Neurobiology, Department of Psychiatry and Behavioral Sciences, Johns Hopkins University School of Medicine, Baltimore, Maryland, United States of America; 3 Department of Biomedical Sciences, Florida State University College of Medicine, Tallahassee, Florida, United States of America; University of Oulu, Finland

## Abstract

The central nervous system (CNS) insults may cause massive demyelination and lead to the release of myelin-associated proteins including its major component myelin basic protein (MBP). MBP is reported to induce glial activation but its effect on neurons is still little known. Here we found that MBP specifically bound to the extracellular surface of the neuronal plasma membrane and induced neurotoxicity *in vitro*. This effect of MBP on neurons was basicity-dependent because the binding was blocked by acidic lipids and competed by other basic proteins. Further studies revealed that MBP induced damage to neuronal membrane integrity and function by depolarizing the resting membrane potential, increasing the permeability to cations and other molecules, and decreasing the membrane fluidity. At last, artificial liposome vesicle assay showed that MBP directly disturbed acidic lipid bilayer and resulted in increased membrane permeability. These results revealed that MBP induces neurotoxicity through its direct interaction with acidic components on the extracellular surface of neuronal membrane, which may suggest a possible contribution of MBP to the pathogenesis in the CNS disorders with myelin damage.

## Introduction

Myelin basic protein (MBP) is the second most abundant protein of the myelin sheath in the central nervous system (CNS) [1]. Because of its essential role in CNS myelin formation and intactness, MBP has been considered as “executive” molecule of myelin [2]. MBP is extremely basic (isoelectric point: ∼10) and carries a highly positive charge [3]. MBP is a member of intrinsically unstructured proteins [4], [5], which characteristically possess a high net charge with low hydrophobicity and maximize intramolecular electrostatic repulsion, leading to an extended structure [6]. As an intrinsically unstructured protein, MBP has sufficient flexibility so that it can interact with a variety of binding partners, including negatively charged lipids [7], [8] and other polyanionic proteins, such as actin filaments [9]–[11], microtubules [12], [13], Ca^2+^-calmodulin [9], [14], tropomyosin [15] and clathrin [16].

Myelin degeneration occurs after insults to CNS, such as chemical toxicity, traumatic brain injury and demyelinating disease [17]–[21]. Demyelination will induce undesirable inflammation [22], [23] and cell death [24]–[30]. Myelin-associated proteins are released after destruction of the intact myelin sheath [31]. Among them, Nogo, myelin-associated glycoprotein, and oligodendrocyte myelin glycoprotein have been identified as inhibitors of neuronal growth by binding to either the Nogo receptor [32], [33] or to a second receptor, PirB [34], [35]. After destruction of the intact myelin sheath, MBP also dissociates from the plasma membrane and acts in a free, membrane-unbound manner in the extracellular matrix [19], [36]. MBP has been well-studied as an antigen to activate immune response throughout the nervous system, which causes immune injury, such as in multiple sclerosis (MS) [1], [37]–[39]. Previous studies have also shown that soluble MBP changes the shape of platelets [40], [41], interrupts artificial membrane and acidic lipid vesicles [42], [43], stimulates proliferation of astrocytes and Schwann cells [44], [45] and depolarizes the neuronal membrane [46]. However, the effect of MBP on neurons is still largely unclear.

It is reported that human eosinophil major basic protein, a cationic protein with high basicity and polycationic amino acids, exerts toxicity on mammalian cells, parasites and bacteria [47]–[49]. Since MBP has similar basicity, we then hypothesize that MBP may induce toxicity on neurons. In the present study, we show that MBP induces selective neurotoxicity *in vitro*, via its direct binding to the extracellular surface of neuronal membrane and subsequent interruption of membrane integrity and function. Depolarization of resting membrane potential (RMP), influxes of cations, leakage of intracellular contents, interruption of membrane fluidity and the disturbance of artificial acidic liposome may altogether account for the mechanism of neurotoxicity induced by MBP.

## Materials and Methods

### Chemicals and antibodies

All animal experiments were approved by the Animal Experimentation Ethics Committee of Zhejiang University and were in complete compliance with the National Institutes of Health Guide for the Care and Use of Laboratory Animals. All surgery was performed under sodium pentobarbital anesthesia, and all efforts were made to minimize suffering. MBP was from Sigma-Aldrich (St. Louis, MO) and Merck (Whitehouse Station, NJ). MBP from Sigma-Aldrich was used unless indicated otherwise. All chemicals were from Sigma-Aldrich unless specified otherwise. Propidium iodide (PI) and 4′-6-diamidino-2-phenylindole (DAPI) were from Beyotime Biotech (Haimen, China). Calcein, Calcein-AM, Fluo-4/AM and FluoZin-1/AM were from Life Technologies (Grand Island, NY). The antibodies used were: rat-anti-MBP (MAB386), mouse-anti-glial fibrillary acidic protein (GFAP) (MAB360) and mouse-anti-GD1a (MAB5606) from Millipore (Billerica, MA); mouse-anti-CD11b (MCA275R) from AbD Serotec (Raleigh, NC); rabbit-anti-Iba1 (019-19741) from Wako Pure Chemical Industries (Osaka, Japan). Neuraminidase from *Clostridium perfringens* (N2876) and FITC-conjugated cholera toxin B subunit (C1655) were from Sigma-Aldrich.

### Primary neuronal cultures

Hippocampal and cortical tissue were harvested from embryonic day 17–19 Sprague Dawley rats of either sex, and then gently chopped and digested in 0.5% trypsin for 13 min at 37°C. Dissociated cells were plated at a density of 0.4×10^4^/cm^2^ in a 35-mm dish with poly-L-lysine-coated coverslips in Neurobasal medium containing 1% horse serum, 0.5 mM glutamine, 1% antibiotic and 2% B27, at 37°C under 5% CO_2_. After 6 h, the medium was replaced with Neurobasal medium containing 0.5 mM glutamine, 1% antibiotic and 2% B27. Subsequently, the culture medium was replaced every 5 d. At 5 days *in vitro* (DIV 5), cytosine arabinoside was added at a final concentration of 2.5 µM. In most experiments, primary hippocampal neurons were used at DIV 10 unless indicated otherwise. For morphological assessment of degeneration in individual neurons, the cultures were transfected at DIV 8 with the pEGFP plasmid (Clontech, Mountain View, CA) using Lipofectamine 2000 (Life Technologies) and used at DIV 10.

### DAPI/PI double-staining

At DIV 10, neurons previously cultured in Neurobasal medium with 2% B27 and 0.5 mM glutamine were washed and transferred to Neurobasal medium only (without any supplement), and then treated with Hank's balanced salt solution (HBSS) or MBP (10–50 µg/mL). MBP was diluted in HBSS before use. Similarly, other cells were washed, transferred and incubated with MBP in the corresponding serum-free medium. After MBP incubation for 24 h, the cells were washed once with extracellular solution (ECS) containing (in mM) 147 NaCl, 2 KCl, 1 MgCl_2_, 2 CaCl_2_, 10 HEPES and 13 glucose, pH 7.4, and then incubated in ECS containing 2 µg/mL propidium iodide (PI, red fluorescence) and 0.1 µg/mL 4′-6-diamidino-2-phenylindole (DAPI, blue fluorescence) for 10 min at 37°C in the dark. Nuclei from cells that were either dead or in late apoptosis were labeled with PI because of disturbed membrane integrity, whereas all nuclei were stained with membrane-permeable DAPI. Afterwards, PI+/DAPI+ and PI−/DAPI+ nuclei were imaged and counted using a confocal microscope (10×/0.4 NA, WD 3.1 mm) (Fluoview FV1000, Olympus, Tokyo, Japan).

### Immunocytochemistry

To determine whether MBP binds to the neuronal extracellular surface, cultured hippocampal neurons at DIV 10 were examined by surface immunostaining for MBP. Briefly, after MBP (10 and 50 µg/mL) treatment for 5 min at 37°C, neurons were carefully washed three times with HBSS, 5 min each time, and fixed in 4% paraformaldehyde (PFA) for 15 min at room temperature. After blocking with 5% bovine serum albumin (BSA) in PBS for 30 min, cells were incubated with primary antibody (MAB386, 1∶200) for 2 h at room temperature and then with corresponding secondary antibody for 1 h at room temperature. The images were acquired using the Fluoview confocal microscope (60×/1.42 NA, WD 0.15 mm, oil-immersion). Neuronal somas were primarily focused on for easier demonstration of MBP surface binding. Primary astrocytes, microglia, oligodendrocytes and endothelial cell line bEND.3 (ATCC, Manassas, VA; CRL-2299) were also subject to this surface-staining protocol.

### Lipid adsorption assay

To assess the effect of acidic lipids on the neuronal surface binding of MBP, neutral lipids [phosphatidylcholine (PC), phosphatidylethanolamine (PE)] and acidic lipids [phosphatidylinositol (PtdIns), phosphatidylserine (PS), phosphatidic acid (PA), monosialoganglioside (GM1), disialoganglioside (GD1a)] were made into liposomes (from 5 to 1000 µM) by sonication in Neurobasal medium. 5 µg of MBP was pre-mixed with 500 µL of above liposome-containing Neurobasal medium for 30 min at room temperature and then this MBP-lipid mixture was used to incubate neurons for 5 min, followed by surface immunostaining for MBP. Neurons free of MBP surface binding indicated the adsorption of MBP by the lipids. To assess the effect of acidic lipids on neuronal mortality, the MBP-phosphatidylinositol (PtdIns) mixture (5 µM PtdIns with 50 µg/mL MBP) was used to incubate neurons for 24 h at 37°C and DAPI/PI double-staining was performed.

### MBP-P2 fraction binding assay

The crude membrane fraction (P2) was extracted from cultured hippocampal neurons using a previous protocol [50]. Briefly, P2 membrane fractions from cultured neurons were prepared by homogenization, low speed centrifugation in 0.32 M sucrose and centrifugation of supernatant at 12000 g for 20 min. The pellet was resuspended in phosphate-buffered saline (PBS) with protease inhibitors [pepstatin 0.7 mg/mL, trypsin inhibitors 0.1 mg/mL, phenylmethylsulfonyl fluoride (PMSF) 1 mM] for use. P2 fraction aliquots, with or without trypsin treatment (0.25% trypsin, 1 h at 37°C), were incubated with 10 µg of MBP for 30 min, washed four times and then collected with centrifugation at 12000 g at 4°C for 10 min. The P2 fraction pellets were denatured in 2×SDS-PAGE loading buffer by boiling at 100°C for 5 min and then subject to SDS-PAGE. After transfer of the proteins onto PVDF membrane, MBP antigen was probed with primary antibody (MAB386, 1∶500) and corresponding secondary antibody. MBP-P2 fraction binding was shown by the presence of MBP bands in P2 fraction pellets. After ECL incubation (GE Healthcare, Piscataway, NJ), the intensities of MBP bands were analyzed using ImageJ 1.46 software (NIH, http://rsbweb.nih.gov/ij/download.html).

### Electrophysiology

Whole-cell current-clamp recording (held at 0 pA) for the measurement of the resting membrane potential (RMP) was performed from the somas of cultured hippocampal neurons in ECS at room temperature, using a MultiClamp 700B amplifier and Digidata 1440A interface (Molecular Devices, Sunnyvale City, CA). The pipette resistance was 3–5 MΩ and the internal solution was (in mM) 130 potassium gluconate, 10 KCl, 10 HEPES, 7 NaCl, 2 MgCl_2_, 0.3 NaGTP, 2.2 Na2ATP, and 0.2 EGTA, pH 7.3. Neurons with RMPs between −70 and −45 mV were selected for experiments. All recordings were performed for 5–10 min, and HBSS or MBP (50 µg/mL) was added into the ECS, close to the pipette, 1 min after recording began. Data were analyzed using Clampfit 10.2 software (Molecular Devices, Sunnyvale, CA).

### Calcium and zinc imaging

For Ca^2+^ imaging, hippocampal neurons were incubated with calcium indicator (1 µM Fluo-4/AM plus 0.02% F-127) in ECS for 30 min. Excessive calcium indicator was washed out, and the cells were further incubated for another 30 min to allow complete de-esterification of the intracellular AM esters. Ca^2+^ transients were elicited by local administration of drugs (50 µg/mL MBP, PRM or BSA) and recorded under the Fluoview confocal microscope (10×/0.4 NA, WD 3.1 mm). Images were acquired in frame-scan mode at a frame rate of 1 Hz. The neuronal soma was selected as the region of interest. Data analysis was performed using the “Time Series Analyzer” plugin of ImageJ 1.46 software. F was the fluorescence intensity at the indicated time point. F0 was the average fluorescence intensity from the first 10 frames in each experiment. Levels of Ca^2+^ increase were shown as F/F0 ratios. Zn^2+^ imaging was performed using the same protocol as for Ca^2+^ imaging except using 3 µM FluoZin-1/AM plus 0.02% F-127 as the zinc indicator and adding 10 µM ZnCl_2_ into the ECS before imaging.

### Plasma membrane damage assay

Calcein (non-permeable; 629 Da) was used to detect damage to the cultured neuronal membrane as previously described [51]. In brief, neuronal cultures were pre-treated with MBP, PRM or BSA for indicated times, and then incubated with calcein (0.32 mM) for 10 min. Then the cultures were washed three times with ECS and the intracellular fluorescence was acquired with the Fluoview confocal microscope (10×/0.4 NA, WD 3.1 mm). Plasma membrane damage was shown by the entry of calcein into living cells. Moreover, cell-permeable molecule calcein-AM was also used to detect whether MBP makes loss of cellular contents. In brief, neuronal cultures were incubated with 1 µM calcein-AM for 30 min, and washed in calcein-AM-free medium to remove excessive dye. Then the cells were treated with MBP (2000 µg/mL), BSA (2000 µg/mL) or PRM (1000 µg/mL), and the intracellular fluorescence was recorded at a frame rate of 1 Hz using Fluoview confocal microscope.

### Fluorescence recovery after photobleaching (FRAP) assay

For FRAP assay on neuronal membrane, the cultures were transfected with pDisplay-GFP plasmid [52] on DIV 9 and used on DIV 10. The FRAP assay was performed as previously described [53] with some modifications, using a confocal microscope (LSM 510 Meta, Zeiss, Oberkochen, Germany) equipped with a 63× phase-contrast plan-apochromat oil objective, NA 1.4. The photobleaching was acquired by 100% power of a 30 mW tunable argon 488-nm laser in a circular area (radius, 30 pixels) for 10 sec, followed by time-lapse imaging recorded at a frame rate of 2.5 Hz. In order to obtain a sufficient speed of scanning, image size was set to 256×256 pixels. Using the Zeiss LSM 510 software version 3.2, the average fluorescence intensity in the bleached area was normalized to 0 at the start of recovery and to 1 at the equilibrium to directly compare the curves from both HBSS and MBP treatment. The diffusion constants were calculated according to equations as previously described [53]. Fluorescence recovery times (t_1/2_) were calculated from the obtained time-courses of fluorescence.

### Artificial liposome vesicle assay

Liposomes were made as previously described [54]. In brief, 20 mg of phosphatidylcholine (PC) or natural-acidic lipid mixtures [50% PC-50% PS; 50% PC-50% PA; 50% PC-50% phosphatidylglycerol (PG); 90% PC-10% PS; 70% PC-30% PS] were dried down from chloroform onto the wall of a thin glass vial. Calcein-containing reconstitution buffer (1 mL with 20 mM Hepes, 100 mM KCl, 40 mM octyl-β-D-glucoside, 50 mM calcein, pH 7.2) was then added to the lipids. After optical clarification by sonication, the calcein-encapsulating liposomes were purified by passing through a G-50 Sephadex column (50–150 µm) and eluted in elution buffer (20 mM Hepes, 100 mM KCl, 0.5 M sucrose, pH 7.2). Calcein fluorescence intensity (excitation at 490 nm and emission at 520 nm) was measured on a SpectraMax M5 plate reader (Molecular Devices, Sunnyvale, CA) in 96-well plate. After 10 min of baseline recording, liposomes were treated with 50 µg/mL BSA or MBP, and then monitored for up to 3 h. Complete calcein release was obtained by lysing liposomes with 10% Triton X-100 and normalized as 100% calcein release. Each treatment was measured in triplicate.

### Statistical analysis

Experiments were repeated at least three times. Data were presented as mean ± SEM. The results were analyzed using Student's t-test, one-way analysis of variance (ANOVA) followed by Dunnett's post-test, or two-way ANOVA followed by Bonferroni's post-test as indicated. Statistical significance was set at P value<0.05.

## Results

### MBP induces neuron-specific toxicity *in vitro*

To investigate the effects of MBP on neurons, we used primary hippocampal neurons because of their physiological importance and the fact that there is extensive demyelination in the hippocampus during injury and neurodegenerative disease [18], [19], [55], [56]. According to the estimated concentration of released MBP after demyelination [57] and previous MBP studies [44], [45], MBP with concentrations ranging from 10 to 50 µg/mL was used in our *in vitro* study. We found that MBP was neurotoxic.

**Figure 1:**
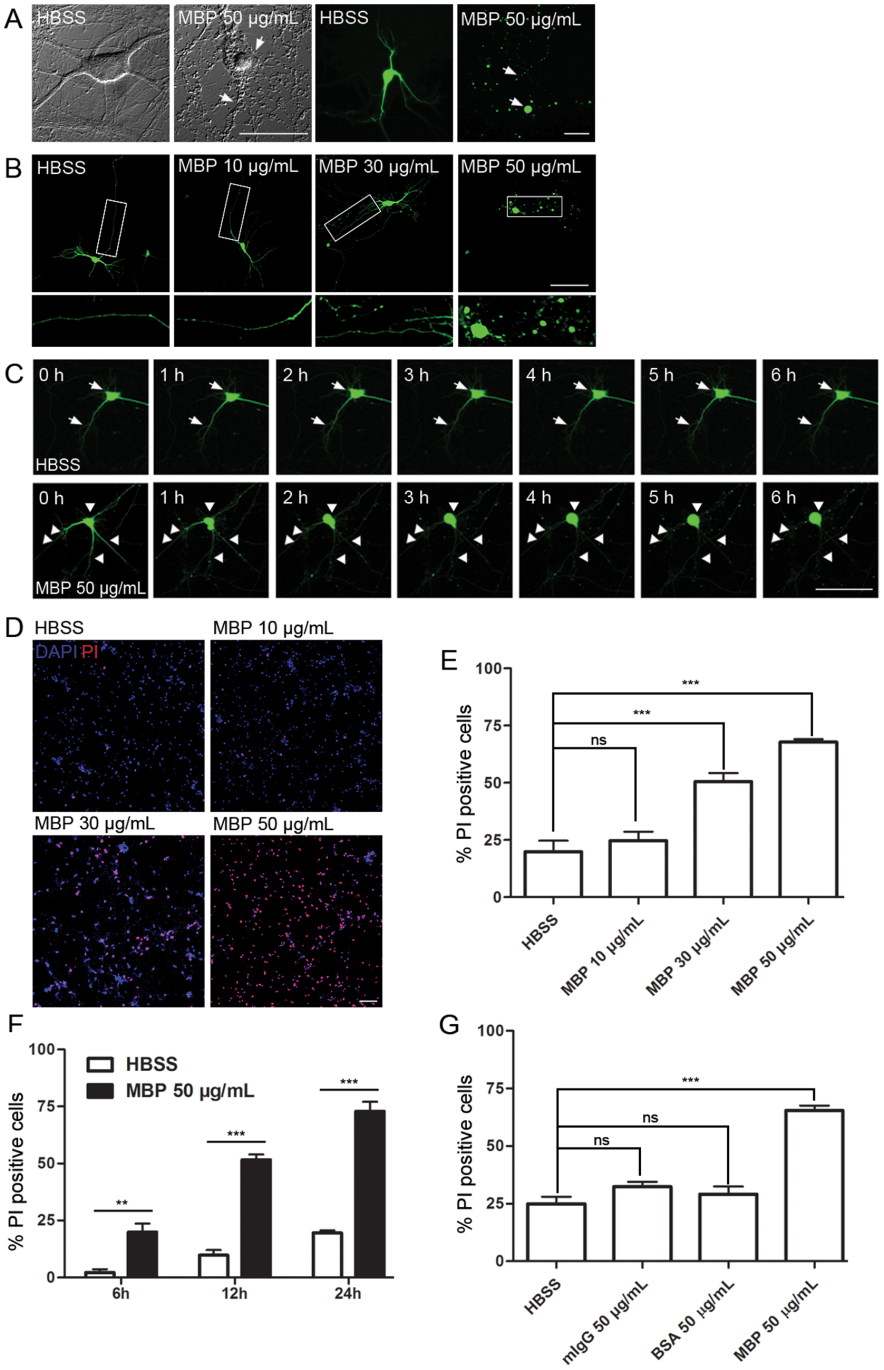
MBP is toxic to primary hippocampal neurons *in vitro*. (A) Morphology of MBP-treated neurons. Primary cultured neurons with or without eGFP overexpression (green) were incubated with 50 µg/mL MBP for 24 h. HBSS was used as control. Degeneration of soma and neurites is indicated with arrows. Scale bar, 50 µm. (B) Neuronal degeneration induced by 24-h MBP incubation at the indicated concentrations. Details are shown in the enlarged images. Scale bar, 100 µm. (C) Time-lapse imaging of neurons incubated with HBSS or 50 µg/mL MBP. Normal morphology is indicated by arrows, and degeneration by arrowheads. Scale bar, 100 µm. (D) Neuronal death induced by 24-h MBP incubation at the indicated concentrations, assessed by DAPI/PI double-staining. Dead cells are PI-positive. Scale bar, 100 µm. (E) Statistical analysis of (D). Incubation with 30 and 50 µg/mL MBP induced cell death (***P<0.001; ns, not significant; one-way ANOVA with Dunnett's post-test; n = 5). (F) Statistical analysis of PI-positive neurons after HBSS or 50 µg/mL MBP incubation for different times (**P<0.01, ***P<0.001; two-way ANOVA with Bonferroni's post-test; 6 h, n = 6; 12 h, n = 10; 24 h, n = 6). (G) Statistical analysis of PI-positive neurons after 24-h incubation with HBSS, mouse IgG (mIgG), BSA or MBP (50 µg/mL each). In contrast to MBP, no cell death was induced by mIgG or BSA (***P<0.001; ns, not significant; one-way ANOVA with Dunnett's post-test; n = 5). Data are mean ± SEM.

First, MBP (50 µg/mL, incubation for 24 h) induced neurotoxicity, featured as typical somatic necrosis and the beaded degradation of neurites (Fig. 1A, arrows). Furthermore, MBP induced neurotoxicity in a dose-dependent manner, where no neurotoxicity was found after MBP treatment at a low concentration (10 µg/mL), while neurotoxicity occurred with > = 30 µg/mL MBP (Fig. 1B). Detailed time-lapse observation revealed that MBP-induced neurotoxicity occurred as early as 1 h after incubation (Fig. 1C, arrowheads) and was progressive.

On the other hand, we assessed neuronal mortality after 24-h MBP treatment using DAPI/PI double-staining, in which DAPI indicates total cells, whereas PI staining shows either dead or late-apoptotic cells. As shown in Fig. 1D–E, control neurons showed basal levels of cell death (∼20%) after 24-h incubation in HBSS, which may result from deprivation of neurotrophic factors during the experiment (see Methods and Materials). MBP at 10 µg/mL did not induce death. However, neurons incubated with 30 or 50 µg/mL MBP for 24 h showed remarkable increase in PI-positive cells. Moreover, MBP-induced death was also time-dependent; a prolonged incubation time was accompanied by increased neuronal death (Fig. 1F). To exclude the possibility that the neurotoxicity of MBP results from its high concentrations (30–50 µg/mL), two control proteins, mouse IgG and BSA at 50 µg/mL, were used to treat neurons for 24 h. No neuronal death was induced by either control proteins (Fig. 1G). Additional experiments showed that MBP obtained from different commercial sources had similar neurotoxicity and primary cortical neurons were also susceptible to MBP (Fig. S1).

**Figure 2:**
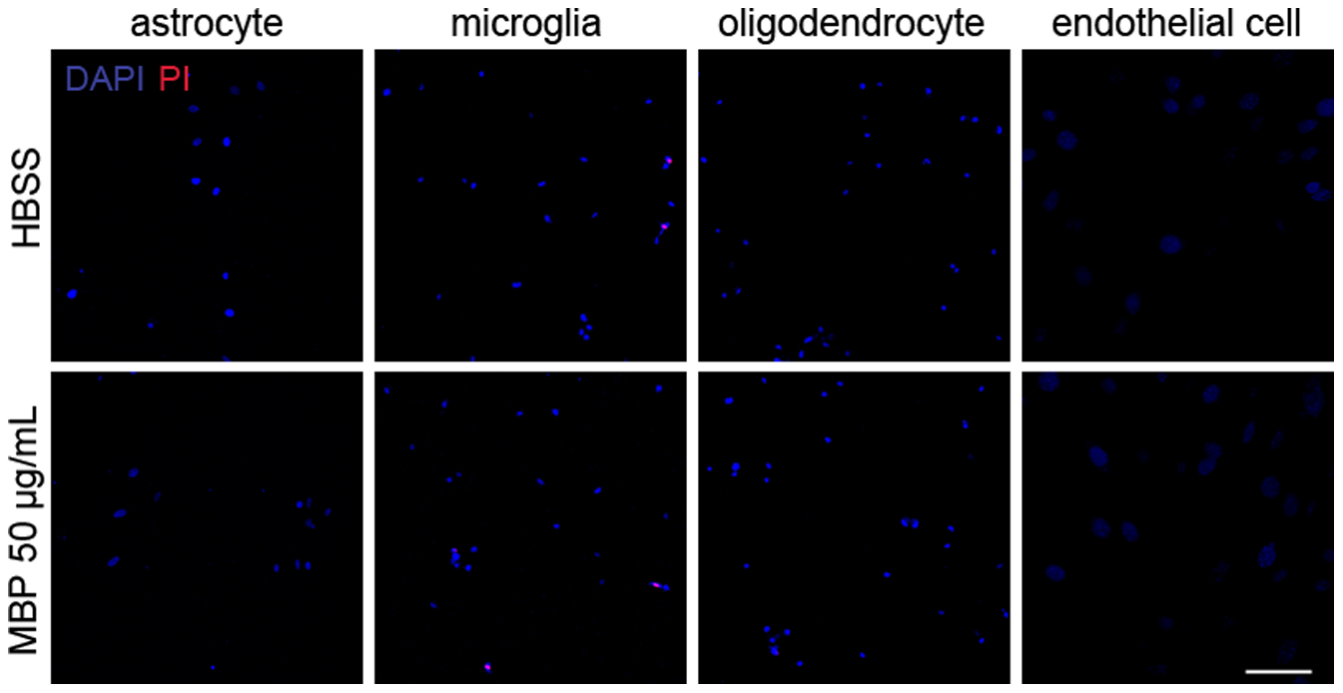
Toxic effect of MBP is neuron-specific. Primary astrocytes, microglia, oligodendrocytes and the endothelial cell line bEND.3 were incubated with HBSS or 50 µg/mL MBP for 24 h. No cell death was indicated by DAPI/PI double-staining. Scale bar, 100 µm.

Besides neurons, the toxic effect of MBP on other cell types in the CNS was also evaluated. Surprisingly, MBP treatment (50 µg/mL, 24 h) was not toxic to primary astrocytes, microglia, oligodendrocytes or an endothelial cell line (Fig. 2). Taken together, these data suggest that MBP induces neuron-specific toxicity.

### MBP binds to the extracellular surface of the neuronal plasma membrane

**Figure 3:**
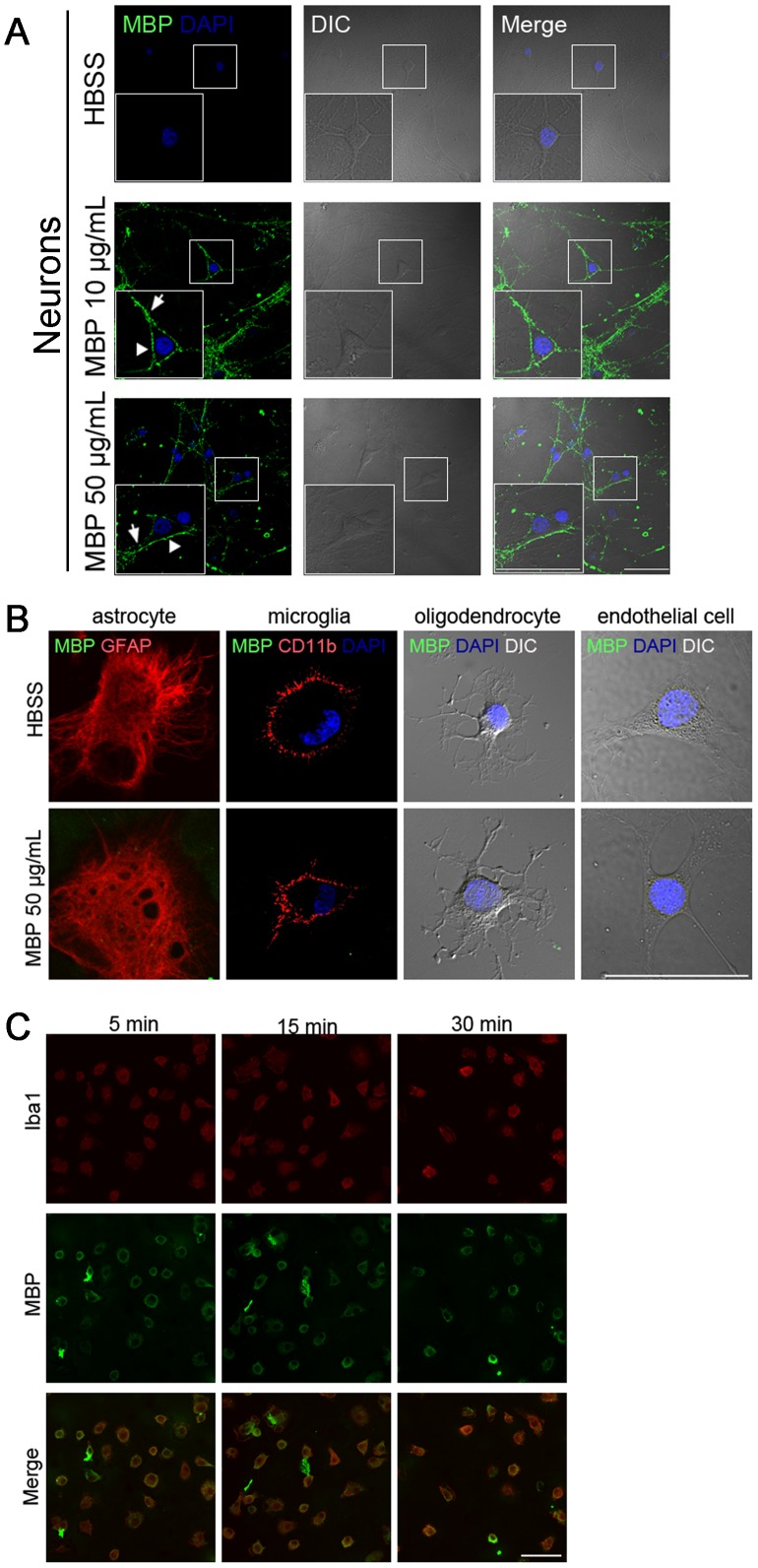
MBP binds to the extracellular surface of neuronal plasma membrane. (A) Neurons were incubated with HBSS, 10 µg/mL or 50 µg/mL MBP for 5 min, and washed for three times with HBSS, 5 min each time. The cells were fixed in 4% paraformaldehyde without permeabilization and stained for surface-bound MBP on plasma membrane (green). Nuclei were indicated by DAPI (blue). General morphology of neurons was observed by differential interference contrast (DIC) microscopy. MBP was shown to bind to the extracellular surface on both soma (arrowheads) and neurites (arrows). Scale bar, 50 µm. (B) Primary astrocytes, microglia, oligodendrocytes and the endothelial cell line bEND.3 were incubated with HBSS or 50 µg/mL MBP for 30 min, washed with HBSS for three times, 5 min each time, and then stained for MBP surface binding (without permeabilization). No surface-bound MBP was detected (green). GFAP and CD11b were used as markers of astrocytes and microglia, respectively (red). Scale bar, 50 µm. (C) Primary microglia were incubated with 50 µg/mL MBP for the indicated times and the intracellular distribution of MBP was shown by staining with permeabilization (green). Iba1 (red) was used as a microglial marker. Scale bar, 50 µm. Similar results were obtained in three independent experiments.

To further explore the mechanism underlying the neuron-specific toxicity of MBP, we performed surface staining for MBP on neuronal cultures after incubation. To technically minimize background from non-specific binding, MBP with low centration (10 µg/mL) was first used to incubate neurons. To our surprise, we found that 5-min incubation of mature neurons with 10 µg/mL MBP resulted in an extensive MBP binding to the surface, not only on the soma (Fig. 3A, arrowhead) but also on all neurites (Fig. 3A, arrow). Incubation with higher MBP concentration (50 µg/mL) gave the same binding pattern (Fig. 3A). MBP also bound to the surface of immature neurons and induced neurotoxicity (Fig. S2). Furthermore, consistent with its specific neurotoxicity, MBP only bound to the extracellular surface of neurons, but not other cell types in the CNS, even if they were incubated for a longer time at the highest concentration (50 µg/mL for 30 min; Fig. 3B). These results show that MBP selectively binds to the neuronal surface.

Besides surface binding, we also asked if MBP can enter neurons by endocytosis. Our data showed no MBP entry into the neuronal cytoplasm (data not shown). This was different from microglia, which took up MBP (Fig. 3C) through endocytosis [58].

### Surface binding and toxic effect of MBP is basicity-dependent

**Figure 4:**
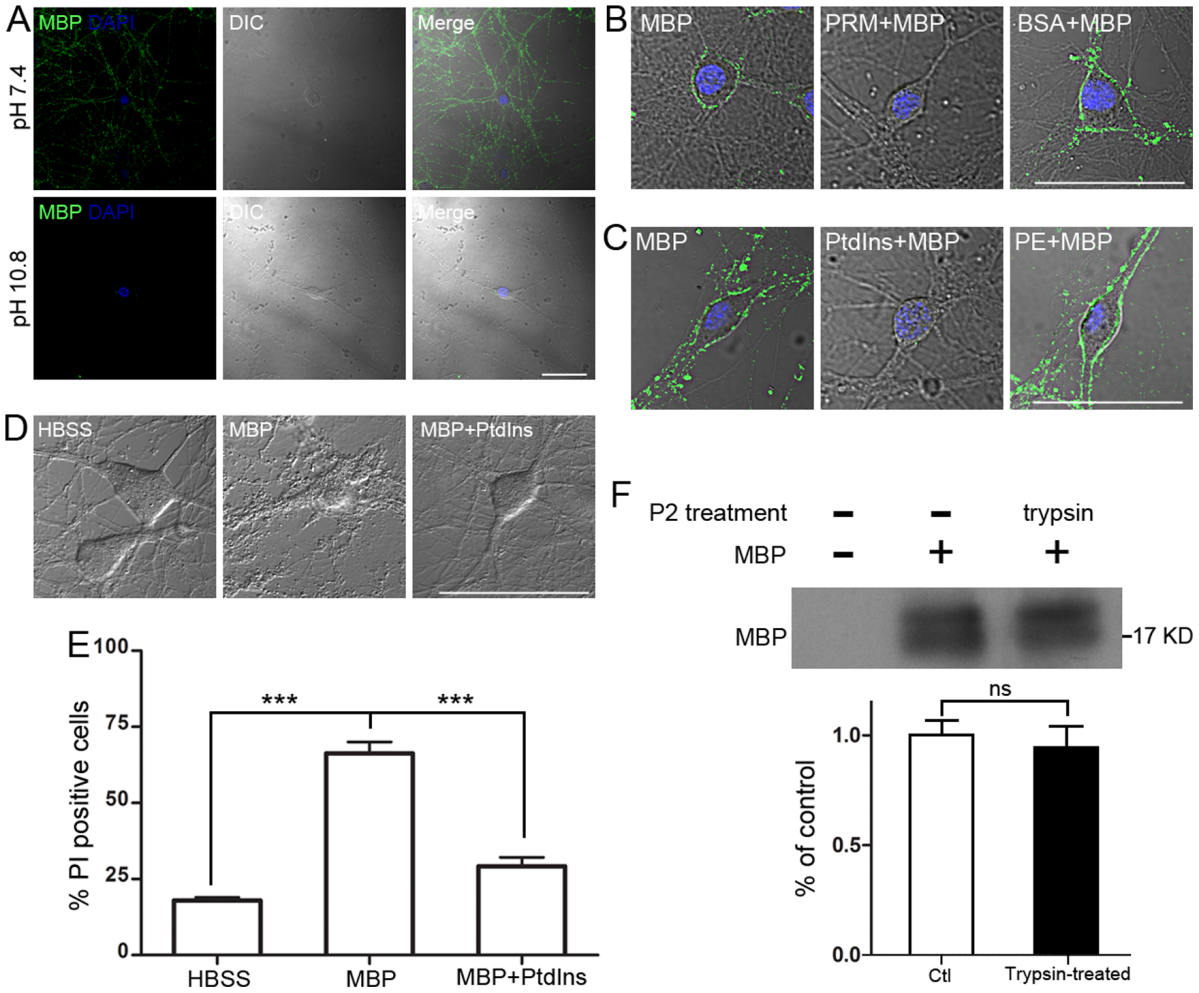
Surface binding and toxicity of MBP rely on polypeptide basicity. (A) MBP surface binding on neurons after 30-min pre-incubation in ECS at pH 7.4 or pH 10.8 (equal to the isoelectric point of MBP) and subsequent treatment with 10 µg/mL MBP for 5 min. MBP surface binding was abolished in ECS at pH 10.8. Scale bar, 50 µm. (B) MBP surface binding on neurons after 30-min pre-incubation with 5 µg/mL PRM or 50 µg/mL BSA and subsequent 5-min treatment with 10 µg/mL MBP. Surface binding of MBP was blocked by pre-incubation of basic protein PRM. Scale bar, 50 µm. (C) MBP surface binding on neurons after 5-min incubation with 10 µg/mL MBP alone, or MBP-lipid mixtures. To obtain the MBP-lipid mixtures, 5 µM phosphatidylinositol (PtdIns, an acidic lipid) or 1000 µM phosphatidylethanolamine (PE, a neutral lipid) were pre-mixed with 10 µg/mL MBP for 30 min. Surface binding of MBP was blocked by the PtdIns pre-mixing. Scale bar, 50 µm. (D) Neurons were incubated with 50 µg/mL MBP alone or a MBP-PtdIns mixture (5 µM PtdIns with 50 µg/mL MBP) for 24 h. No neuronal degeneration was found by morphology with MBP-PtdIns treatment. Scale bar, 50 µm. (E) Statistical analysis of neuronal death by DAPI/PI double-staining after treatment in (D) (***P<0.001; one-way ANOVA with Dunnett's post-test; n = 5). (F) Binding of MBP with the neuronal P2 fraction. The P2 fraction, with or without trypsin pre-treatment (0.25%, 37°C for 1 h), was incubated with 10 µg of MBP for 30 min, washed and then assessed for MBP association by western blot. MBP was shown to bind with the P2 fraction independent of trypsinization (ns, not significant; unpaired, two-tailed t-test; n = 3). Data are mean ± SEM.

It is well-known that in the intact myelin sheath, MBP interacts with acidic lipids located on the inner surface of the plasma membrane through MBP's basic residues [3]. We then hypothesized that MBP binds to neurons through electrostatic interaction. Since MBP is a basic protein with an isoelectric point of 10.8, according to our hypothesis, at a pH value of 10.8 MBP is neutral and would lose the ability to bind to acidic lipids on the neuronal surface. To test our hypothesis, we performed MBP surface staining at pH 10.8 and found that it did not bind to the neuronal surface (Fig. 4A). It is notable that MBP undergoes self-assembly in a basic solution [59]. Moreover, we used another typical basic protein, protamine (PRM), whose isoelectric point is ∼12–13 [60], and found that pre-incubation of neurons with 5 µg/mL PRM for 30 min blocked all MBP surface binding (Fig. 4B). PRM also induced neurotoxicity on neurons (Fig. S3). However, pre-incubation with 50 µg/mL BSA (isoelectric point: ∼4.7) did not block any subsequent MBP surface binding (Fig. 4B). Next, we used insoluble liposomes to pre-treat MBP before its incubation with neurons, and accordingly found that MBP neutralized by acidic liposomes failed to bind to neurons, but pre-treatment of MBP with neutral liposomes had no such effect (Table 1). Among the acidic lipids we tested, phosphatidylinositol (PtdIns) was the most efficient in blocking MBP surface binding (Table 1, Fig. 4C) and phosphatidic acid showed the weakest blocking efficiency (Table 1). However, all the neutral lipids, including phosphatidylethanolamine (PE), had no blocking ability (Table 1, Fig. 4C). Consistent with the surface binding, further neurotoxic assay revealed that pre-treated MBP with PtdIns (5 µM PtdIns with 50 µg/mL MBP) did not induce significant neuronal degeneration or death (Fig. 4D–E), indicating that MBP induces neurotoxicity through extensive binding to neuronal membrane. So far, unlike other myelin-associated proteins [35], no any specific membrane receptor for MBP has been identified. To reveal more details about the binding partners of MBP on the neuronal surface, we hydrolyzed all the protein receptors on the neuronal membrane by trypsinizing the crude membrane fraction (P2) from neurons (0.25% trypsin, 1 h at 37°C), and then examined the association of MBP with this fraction. Compared with control, the binding of MBP with the P2 fraction was not affected by trypsinization of membrane protein (Fig. 4F). In addition, heat-inactivated MBP still bound to the neuronal surface and induced toxicity (Fig. S4). Taken together, these data suggest that MBP-induced neurotoxicity is basicity-dependent and may not be directly related to membrane proteins.

**Table 1:** Neuronal surface binding of MBP (10 µg/ml, 5 min) is abolished by MBP-acidic lipid pre-mixing.

Lipid (pre-mixed with MBP)	Concentration (µM)					
	5	25	100	250	500	1000
PtdIns (acidic)	No^a^	No	No	No	——c	——
PS (acidic)	Yes^b^	No	No	No	——	——
PA (acidic)	——	Yes	Yes	Yes	Yes	No
GM1 (acidic)	——	——	——	No	No	——
GD1a (acidic)	——	——	No	No	——	——
PC (neutral)	——	——	——	Yes	Yes	Yes
PE (neutral)	——	——	——	Yes	Yes	Yes

MBP was pre-mixed with various lipids, the mixture was used for neuronal incubation, and subsequent MBP surface staining was performed to assess MBP surface binding (see Materials and Methods for details). PtdIns: phosphatidylinositol; PS: phosphatidylserine; PA: phosphatidic acid; GM1: monosialoganglioside; GD1a: disialoganglioside; PC: phosphatidylcholine; PE: phosphatidylethanolamine.

a : No MBP surface staining observed.

b : MBP surface binding observed.

c : Not done.

### MBP interrupts functions of the neuronal plasma membrane

**Figure 5:**
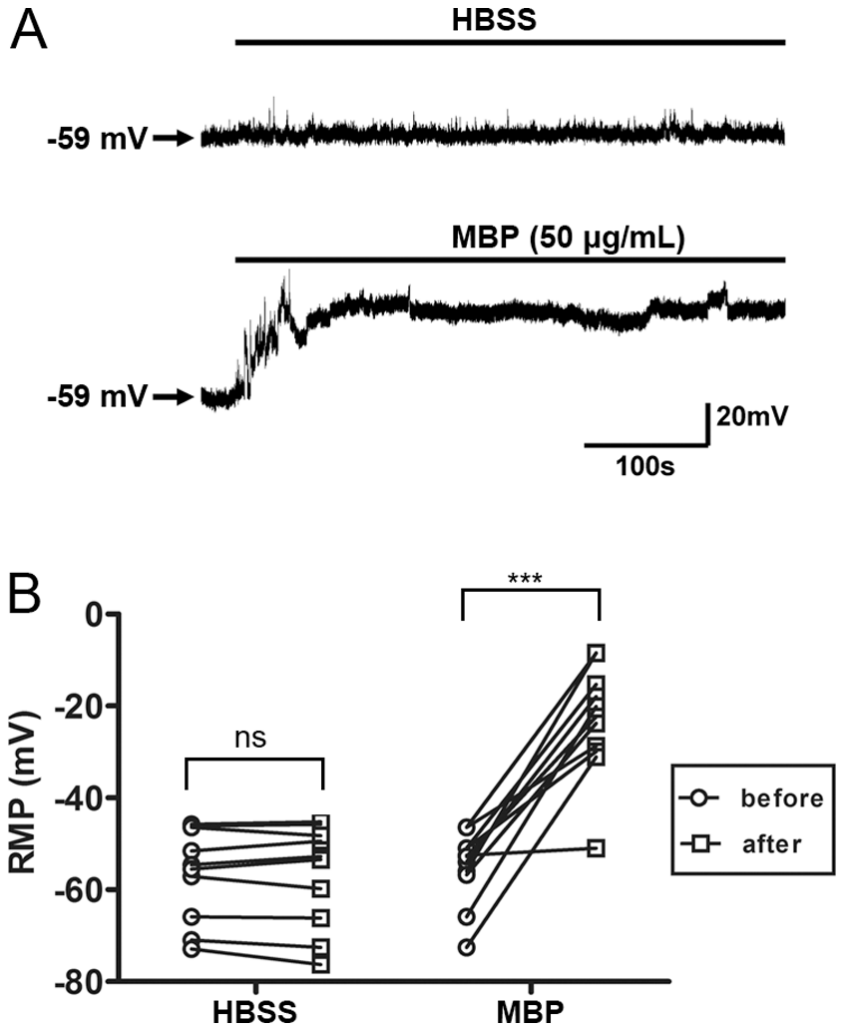
MBP depolarizes the resting membrane potential. (A) Representative neuronal resting membrane potentials (RMPs) recorded before and after HBSS or 50 µg/mL MBP treatment in current-clamp mode. (B) Statistical analysis of RMP changes. Compared to those in HBSS treatment (ns, not significant; paired, two-tailed t-test; n = 10), neuronal RMPs were depolarized after MBP treatment (***P<0.001; paired, two-tailed t-test; n = 11).

**Figure 6:**
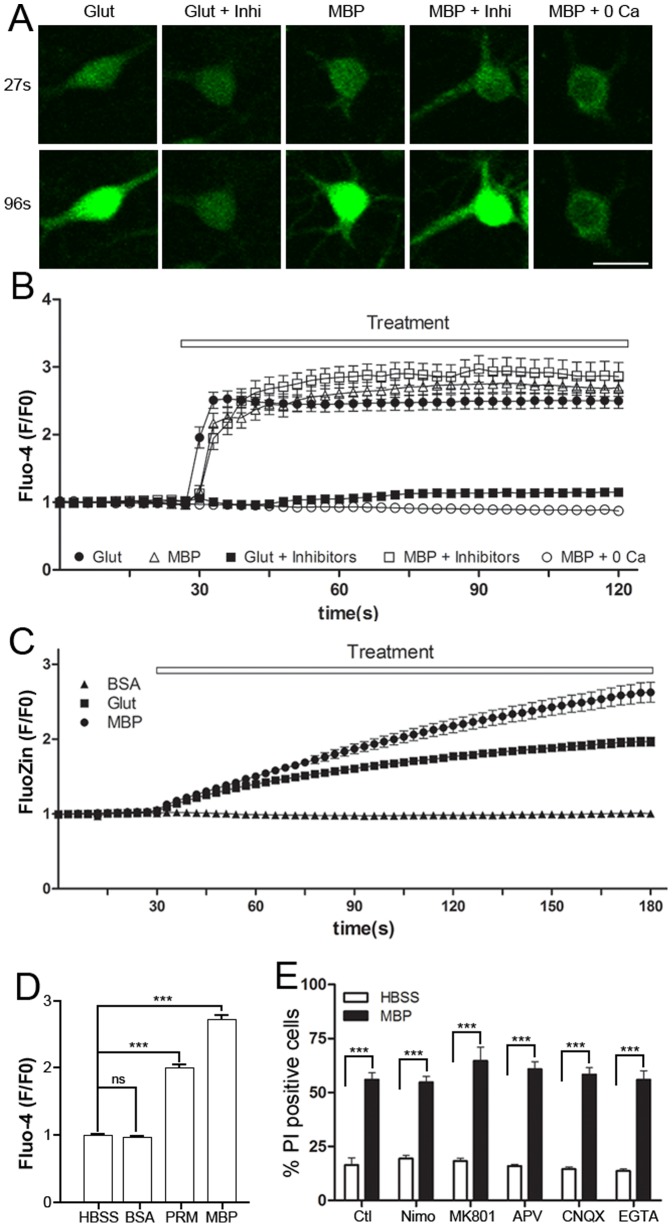
MBP induces cation influx. (A) Ca^2+^ influx in neurons measured by Fluo-4 fluorescence. Fluo-4-loaded neurons were treated as indicated and monitored for 90 sec after 30 sec of baseline recording. The representative images are shown before (27 sec) and after (96 sec) drug administration. Scale bar, 25 µm. Intracellular Ca^2+^ was increased by 600 µM glutamate (n = 20) or 50 µg/mL MBP (n = 21). “Glut+Inhi” (n = 18) and “MBP+Inhi” (n = 21) represent the presence of APV (100 µM), CNQX (20 µM) and nimodipine (10 µM) during treatment. “MBP+0 Ca” (n = 17) represents neurons incubated with MBP in a Ca^2+^-free solution. (B) Time course of the Ca^2+^ increase (Fluo-4 F/F0) in different conditions. Note that a plateau was reached shortly after treatment. (C) Time course of Zn^2+^ influx (FluoZin F/F0) in neurons treated with 50 µg/mL BSA (n = 38), 50 µg/mL MBP (n = 40) or 600 µM glutamate (n = 26) in the presence of 10 µM external Zn^2+^. (D) Statistical analysis of Ca^2+^ increase after treatment with HBSS (n = 17), 50 µg/mL MBP (n = 24), PRM (n = 23) or BSA (n = 21) (***P<0.001; ns, not significant; one-way ANOVA with Dunnett's post-test). (E) Statistical analysis of neuronal death by DAPI/PI double-staining after 24-h incubation with 50 µg/mL MBP in the presence of APV (100 µM), MK801 (40 µM), CNQX (20 µM), nimodipine (10 µM) or EGTA (1 mM) (***P<0.001; unpaired, two-tailed t-test; n = 4). Data are mean ± SEM.

The extensive MBP binding on the neuronal surface inspired us to investigate potential changes in plasma membrane function. Normally functioning neuronal plasma membrane is capable of maintaining its normal resting membrane potential (RMP), permeability and fluidity [61]. Previous study has shown that MBP depolarizes the neuronal membrane, and this effect cannot be blocked by the inhibitors of Na^+^ or Ca^2+^ channels, even in low-Na^+^ and -Cl^−^ solutions [46]. We also obtained the same results in cultured hippocampal neurons. Compared with control, MBP significantly depolarized the RMP (Fig. 5A–B). It is notable that MBP depolarized membrane potential in about 60 seconds and then maintained it at about −20 mV. Because the depolarization induced by MBP was not due to the flow of a specific ion [46], we speculated that the surface binding of MBP may induce depolarization by causing a non-selective flow of ions. To test this, Ca^2+^ and Zn^2+^ imaging were performed on cultured neurons, and several inhibitors of common calcium channels were used in calcium imaging to see if these channels are involved in calcium influx induced by MBP. We found that, like glutamate, MBP incubation significantly increased the intracellular Ca^2+^ ([Ca^2+^]_i_); however, in contrast to glutamate, this effect was not blocked by the antagonist of N-methyl-D-aspartate receptors (NMDARs) [R-2-amino-5-phosphonopentanoate (APV), 100 µM], antagonist of α-amino-3-hydroxy-5-methyl-4-isoxazolepropionic acid receptors (AMPARs) [6-cyano-7-nitroquinoxaline-2,3-dione (CNQX), 20 µM], and blocker of voltage-gated Ca^2+^ channels (nimodipine, 10 µM) (Fig. 6A–B). In addition, Zn^2+^ imaging performed in ECS with 10 µM external Zn^2+^ also showed that MBP, but not the control protein BSA, significantly increased intracellular Zn^2+^ ([Zn^2+^]_i_) (Fig. 6C). Another basic protein, PRM, was also shown to induce an increase in [Ca^2+^]_i_, while BSA did not (Fig. 6D).

**Figure 7:**
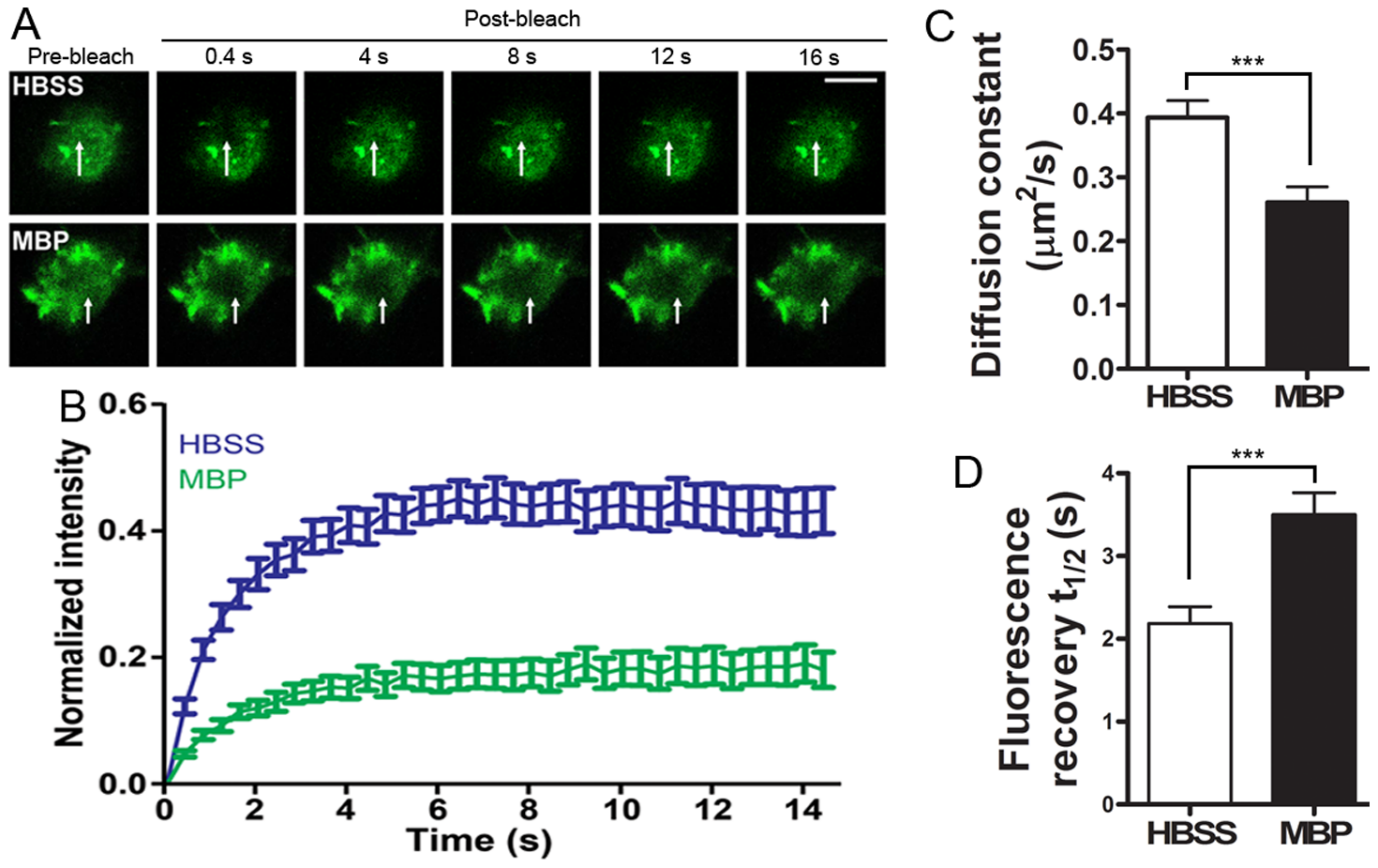
MBP interferes with the fluidity of the neuronal plasma membrane. (A) Hippocampal neurons expressing pDisplay-GFP plasmid were treated with HBSS (n = 31) or 50 µg/mL MBP (n = 41) and then subject to FRAP assays. Representative images showing membrane-bound GFP were acquired before bleaching (pre-bleach) and 0.4, 4, 8, 12 and 16 sec after bleaching (post-bleach), with arrows indicating the photobleached areas. Scale bar, 5 µm. (B) Recovery curves measured by GFP intensity in (A). (C) Diffusion constant of the FRAP curve (***P<0.001; unpaired, two-tailed t-test). (D) Fluorescence recovery time t_1/2_ of the FRAP curve (***P<0.001; unpaired, two-tailed t-test). Data are mean ± SEM.

Based on the non-selective flow of ions, further neuronal mortality assays revealed that MBP-induced neuronal death was independent of any antagonists against NMDARs (MK801, 40 µM; APV, 100 µM), AMPARs (CNQX, 20 µM) and voltage-gated Ca^2+^ channels (nimodipine, 10 µM), or the absence of external Ca^2+^ (EGTA, 1 mM) (Fig. 6E). We also investigated the effect of MBP binding on membrane fluidity by performing FRAP assays using pDisplay-GFP, a membrane-bound reporter exclusively expressed on the extracellular surface of the plasma membrane [52]. Compared to control (0.4315±0.0358, n = 31), neurons treated with MBP had attenuated fluorescence recovery (0.1810±0.0279, n = 41) in a 16-sec time frame (Fig. 7A–B), along with limited diffusion mobility (Fig. 7C) and a longer recovery time (Fig. 7D). All these data indicate that MBP interrupts the normal functions of the neuronal plasma membrane.

### MBP increases permeability of the neuronal membrane

**Figure 8:**
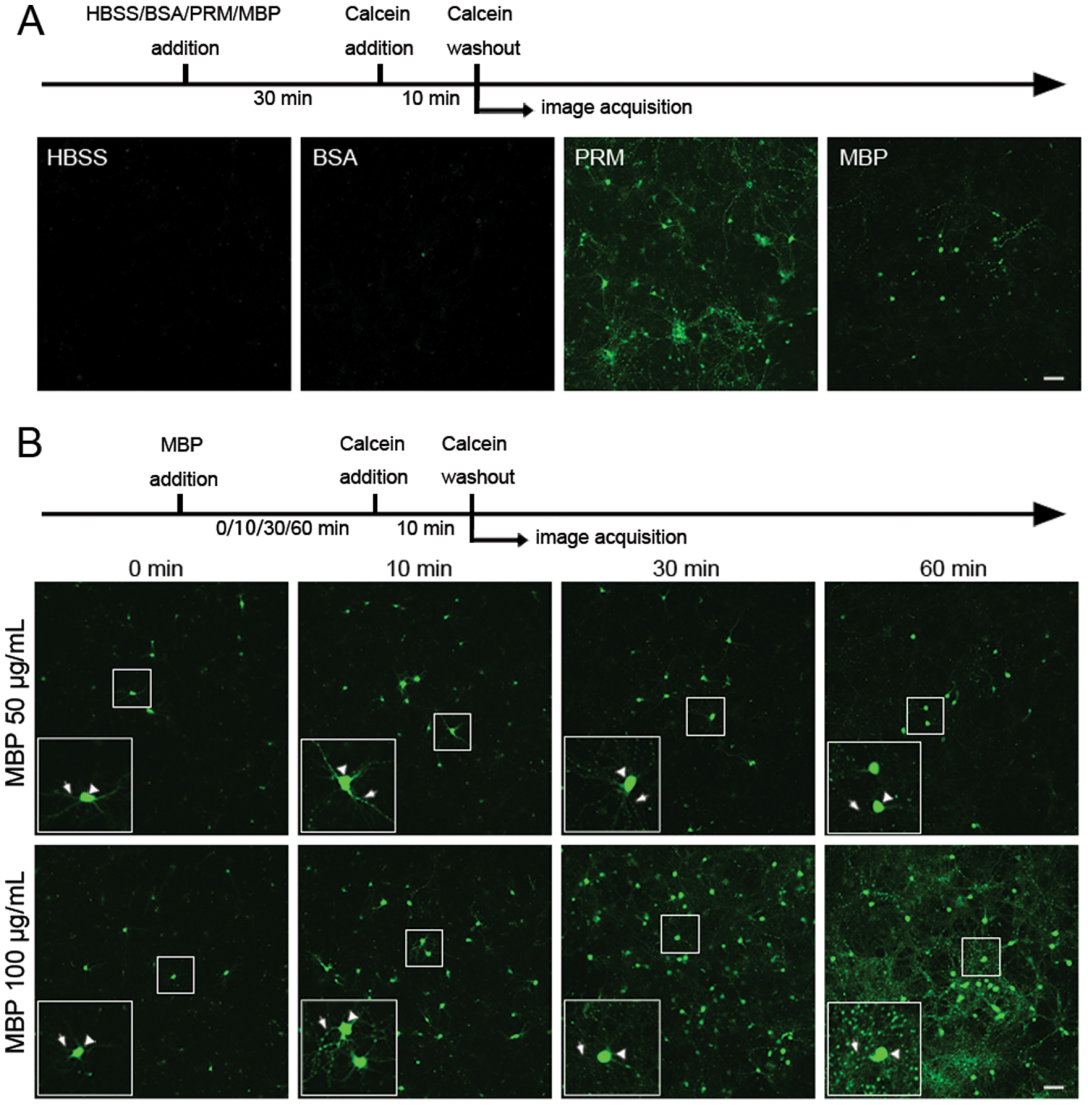
MBP damages neuronal membrane integrity. (A) Damage to the neuronal plasma membrane was assessed by the intracellular presence of living cell-impermeable calcein. Neurons were pre-treated with HBSS, 50 µg/mL BSA, PRM or MBP for 30 min and then incubated with calcein for additional 10 min. Details are shown in the timeline. Scale bar, 100 µm. (B) Plasma membrane damage after pre-treatment with 50 or 100 µg/mL MBP for 0, 10, 30 and 60 min. Scale bar, 100 µm. Note that neurons were still treated with MBP for 10 min in the calcein step of the “0 minute” group. Similar results were obtained in three independent experiments.

**Figure 9:**
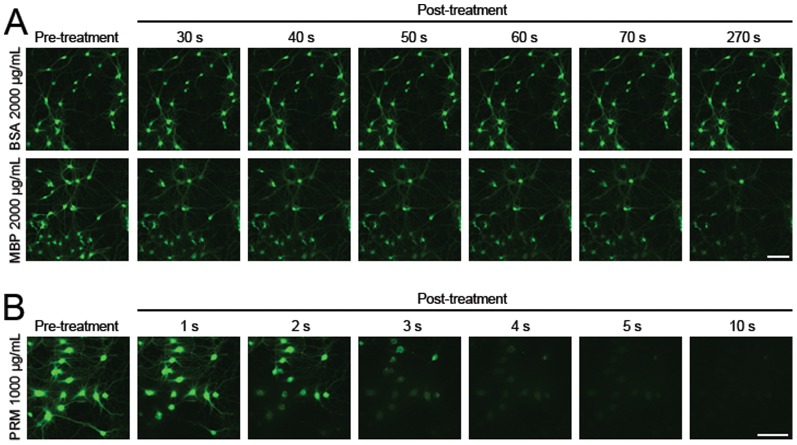
MBP at a high concentration induces the loss of cellular contents. (A) and (B) Representative images of hippocampal neurons pre-loaded with calcein-AM (cell-permeable calcein), before and after 2000 µg/mL BSA, 2000 µg/mL MBP or 1000 µg/mL PRM incubation. MBP and PRM incubation induced significant loss of intracellularly loaded calcein. Scale bar, 100 µm.

Besides MBP-induced ion-scaled influx, we also investigated the possible flow of large molecules through the plasma membrane, which would indicate the damage of the plasma membrane. Calcein is a living cell-impermeable molecule (629 Da) and enters cytoplasm after membrane damage, which is used as a marker of membrane permeability at the molecular scale [51]. We found that the permeability increased after 30-min incubation with 50 µg/mL MBP or PRM, as shown by heterogeneous calcein uptake into the cytoplasm (Fig. 8A). Like the Ca^2+^ and Zn^2+^ imaging, no calcein uptake occurred after BSA treatment (50 µg/mL). To explore in detail how MBP permeabilizes the neuronal plasma membrane, calcein uptake was examined after 50 or 100 µg/mL MBP incubation for different times. We found that MBP increased the membrane permeability with time and concentration (Fig. 8B). Besides influx of extracellular materials into cells, there may also be loss of cellular contents after damage to membrane permeability. Therefore, we used calcein-AM to detect whether MBP induces loss of cellular contents. Unlike calcein, calcein-AM is a cell-permeable molecule and readily enters living cells, and loss of intracellular calcein-AM signal indicates loss of cellular contents. To make the phenomenon more obvious, we used relatively high concentration of MBP (2000 µg/mL) and found that pre-loaded calcein-AM began to leak from neurons in a 30-second time when neurons were treated with MBP (2000 µg/mL), but the same concentration of BSA had no such effect (Fig. 9A). We also found that lower concentration of PRM (1000 µg/mL) even induced severer effect than MBP did (Fig. 9B). Although concentration of MBP used here may be out of physiological range, the result is still useful to consolidate the conclusion that MBP damages the neuronal membrane.

### MBP directly disrupts integrity of artificial lipid bilayer membrane

**Figure 10:**
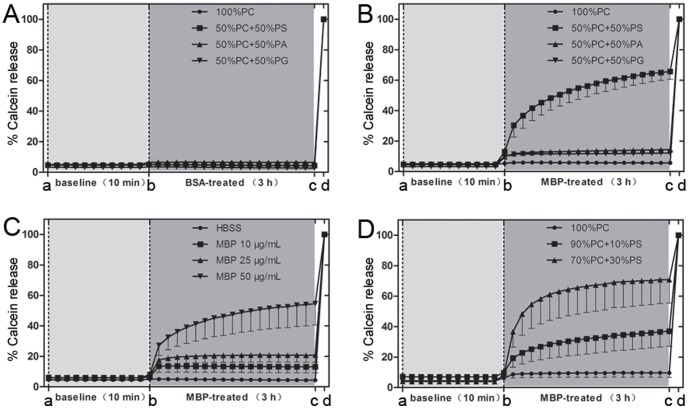
MBP directly disrupts the integrity of the lipid bilayer *in vitro*. (A) and (B) Release of calcein from calcein-loaded liposomes after 50 µg/mL BSA or MBP treatment. Liposomes were prepared from the indicated components. 10% Triton X-100 (from c to d) was used to induce 100% calcein release. (C) Effect of different concentrations of MBP on calcein release from calcein-loaded liposomes containing a 1∶1 mass ratio of PC∶PS. (D) Effect of MBP treatment (50 µg/mL) on calcein release from liposomes containing different mass ratios (7∶3 or 9∶1) of PC∶PS. Data are from three independent experiments and indicated as mean ± SEM.

To further investigate the mechanism of MBP-induced damage to the neuronal membrane, we performed artificial liposome vesicle assay (Fig. 10). Calcein, when highly concentrated in liposomes, is self-quenched and shows no fluorescence; however, released calcein from liposomes is fluorescent due to decrease of its concentration. Therefore the calcein fluorescence intensity was a marker of calcein release and used as a measurement of damage to the artificial liposomes. We encapsulated calcein into liposomes made of different lipids, treated liposomes with MBP (50 µg/mL) and then evaluated the damage of MBP to liposomes. We found that MBP destroyed acidic liposome vesicles rather than neutral liposome vesicles as indicated by increased calcein release (Fig. 10A–B). In addition, the effect of MBP on acidic liposome vesicles was dose-dependent (Fig. 10C). At last, with the increase in proportion of acidic phospholipid, the effect of MBP became stronger (Fig. 10D). These data indicate that a direct interaction between MBP and acidic lipid on neuronal surface may be responsible for the damage of MBP to the neuronal membrane.

## Discussion

As an abundant protein in the CNS and an essential component of myelin sheath, MBP has been well-studied since its first report. Lots of literatures have elucidated the fundamental function of MBP in oligodendrocyte and myelin sheath [9], [15], [16], [62]–[64]. There are also extensive studies demonstrating immune-activating effect of MBP on immune system during demyelinating disease [37], [38]. MBP is also reported to induce the proliferation of cultured Schwann cells and astrocytes [44], [45]. In contrast, we found in this study that MBP induces neuronal damage. Moreover, to the best of our knowledge, both neurotoxicity induced by most of the biological molecules and growth inhibitory effect from other well-known myelin-associated proteins are commonly under the canonical regulation of finely-tuned signaling pathways, in which receptors on extracellular surface sense the stimuli, activate intracellular signaling partners and induce biological effect through complicated cascades [34], [35], [65]–[69]. However, neurotoxicity induced by MBP differentially features rapid and dramatic disturbance of membrane permeability and fluidity after MBP directly targets neuronal plasma membrane (Fig. 6, 7, 8, 9) and is basicity-dependent and receptor-independent (Fig. 4).

Although whether such neuron-selective toxicity by MBP observed *in vitro* also occurs *in vivo* remains unsolved, the possibility is suggested by following observations. First, the concentrations of MBP used *in vitro* may reflect the actual release of soluble MBP after demyelination *in vivo*. MBP is abundant in the brain, comprising 30% of total myelin protein [1]. The overall concentration of soluble MBP in the brain lysate is reported to be as high as 800 µg/mL in mouse [70] and 1600 µg/mL in rat [71]. Westall et al. discussed the amount of released MBP accumulating in the vicinity of myelin and believed a minimal local concentration of 220 µg per gram of myelin can be reached [57]. Our study showed that MBP induced neurotoxicity at concentrations from 30 to 50 µg/mL, similar to those used in other *in vitro* studies on MBP functions, which are usually 20–200 µg/mL [44], [45], [72]–[74]. While a reliable *in vivo* measurement of concentration of soluble MBP is difficult to be achieved, these studies make it reasonable to correlate the concentrations of MBP used in our *in vitro* study with the real concentration of membrane-unbound MBP in demyelinating foci *in vivo*. Second, massive hippocampal demyelination is associated with loss of hippocampal neurons in cuprizone-induced mouse model of demyelination [26]. Cuprizone leads to loss of oligodendrocytes, producing abundant myelin debris in mice [26], [75]–[78], without damage to blood brain barrier or activation of T cells [77], [79]. Moreover, using amyloid precursor protein (APP) as a marker, acute and massive axonal damage is observed during the stage of active demyelination in multiple sclerosis (MS) [80], [81]. Both early and late active demyelination lesions are characterized by infiltration of MBP-positive macrophages [82], indicating release of MBP in demyelinating lesions. Therefore we speculate that neurotoxicity of MBP *in vitro* may also occur during neuronal damage following demyelination *in vivo*.

Our study showed that the normal functions of neuronal plasma membrane were interrupted during MBP-induced neurotoxicity. Both the lipid bilayer and membrane receptors are involved in the maintenance of normal membrane function. The fact that MBP still bound to the membrane fraction in the presence of proteolysis suggests that membrane receptors are unlikely to be involved in MBP-induced neuronal damage (Fig. 4F). It is reported that eosinophil granule major basic protein, with the isoelectric point similar to that of MBP, interacts with synthetic acidic lipid bilayers resulting in the fusion and lysis of liposomes [49]. These studies, along with our finding that MBP induces neurotoxicity in a basicity-dependent manner (Fig. 4), suggest that MBP functions through direct interaction with acidic components on the neuronal plasma membrane. Accordingly, we also found that MBP directly lyses acidic lipid-containing liposomes, whereas liposomes composed of neutral lipids were not affected (Fig. 10). Another piece of evidence to support this idea is that, in our study, PRM behaved like MBP (Fig. 6D, 8A, 9B). These results demonstrate that, although there are still potential membrane receptors of MBP [36], [44], direct interaction between MBP and acidic components on the plasma membrane may be mainly responsible for MBP-induced dysfunction. Therefore, MBP affects neurons in a quite different manner from other myelin-associated proteins, where classic ligand-receptor interactions and signal transduction are involved [32]–[35]. This idea can also be supported by other studies. Previous studies showed that the shape change of blood platelets induced by MBP can be attenuated by acidic mucopolysaccharide heparin [40], [41], which is similar with the protective effect of acidic phospholipids on MBP-induced neurotoxicity (Fig. 4D–E). These studies also revealed that MBP-induced platelet shape change is not inhibited by antagonists of 5-hydroxy-tryptamine receptors [40], [41], which is similar with the fact that antagonists and blockers of multiple channels failed to rescue MBP-induced neurotoxicity (Fig. 6E). Moreover, Gahwiler et al. reported that the depolarization of RMP induced by MBP cannot be blocked by any selected ion channel inhibitors [46], which is similar with our finding that non-selective cation influx occurs after binding of MBP to neuronal extracellular surface (Fig. 6B–C).

If acidic components are the main interactors with MBP, then the next question is the identity of specific acidic components as binding partners. Lipids on the plasma membrane are of various kinds, such as glycerophospholipids, sphingolipids and gangliosides [83]. Among them, acidic glycerophospholipids, such as phosphatidic acid, phosphatidylserine, phosphatidylglycerol, PtdIns, as well as gangliosides that have sialic acids, may be candidate binding partners of MBP. Acidic glycerophospholipids are extensively distributed in nearly all kinds of cells. However, our results showing that MBP only acts on neurons (Fig. 2) suggest that the MBP-binding lipids may be specifically located on neurons. Moreover, it is well-known that the distribution of glycerophospholipids across membrane is asymmetric [84], [85]. The outer leaflets of membrane are enriched in zwitterionic glycerophospholipids such as phosphatidylcholine, whereas most acidic glycerophospholipids such as phosphatidylserine are located in the inner leaflets [86]. Hence, we speculate that glycerophospholipids are less likely to be the main binding partners of MBP. Previous studies showing that gangliosides are enriched in neurons and interact with basic proteins [87] and that the ganglioside GM1 interacts with MBP on Schwann cells [44] suggest a potential role of ganglioside as a binding partner of MBP on the neuronal plasma membrane. Cholera toxin B (CTB) specifically binds to GM1 through sialic acid [88]. We found that pre-incubation of MBP effectively blocked binding of CTB on neuronal surface, suggesting MBP may bind to GM1 on neurons (Fig. S5A). However, selective blockage of GM1 by CTB alone failed to significantly abolish MBP surface binding (Fig. S5B) or attenuate its neurotoxicity (Fig. S5C), indicating other binding partners may also be involved. We removed terminal sialic acids on neuronal surface by pre-incubation of neuraminidase (Fig. S5D) [89] and observed drastic decrease of MBP surface binding (Fig. S5E). This result suggests that sialic acid may be the main binding partner of MBP on neuronal surface. However, the possibility still exists that other uncommon acidic components, such as acidic carbohydrate chains, which are specifically located on the outer leaflets of neuronal plasma membrane, mediate MBP-induced neurotoxicity.

## Supporting Information

Figure S1 **Further confirmation of MBP-induced neurotoxicity.** (A) Neurodegeneration of hippocampal neurons after 24-h incubation with 50 µg/mL MBP purchased from either Sigma or Merck. Scale bar, 50 µm. (B) Statistical analysis of PI-positive neurons after 24-h incubation with 50 µg/mL MBP from either Sigma or Merck (***P<0.001, one-way ANOVA with Dunnett's post-test, n = 5). (C) Statistical analysis of PI-positive cortical neurons after 24-h incubation with 50 µg/mL MBP (***P<0.001; unpaired, two-tailed t-test, n = 3). Data are mean ± SEM.
(TIF)

Figure S2 **MBP-induced neurotoxicity is independent of neuronal development** ***in vitro*** **.** (A) Morphology of hippocampal neurons at DIV 1, 2 and 5 after incubation with 50 µg/mL MBP for 24 h. (B) Surface binding of MBP on neurons at DIV 1, 2 and 5 after incubation with 10 µg/mL MBP for 5 min. Scale bar, 100 µm.
(TIF)

Figure S3 **PRM induces neurotoxicity.** (A) Neurodegeneration of hippocampal neurons after 24-h incubation with HBSS, 5 µg/mL or 50 µg/mL PRM. Scale bar, 50 µm. (B) Statistical analysis of PI-positive neurons after 24-h incubation with HBSS, 5 µg/mL or 50 µg/mL PRM. (***P<0.001, ns, not significant; one-way ANOVA with Dunnett's post-test, n = 4). Data are mean ± SEM.
(TIF)

Figure S4 **Neurotoxicity induced by heat-inactivated MBP.** (A) Hippocampal neurons were incubated with 10 µg/mL native or heat-inactivated MBP (30 min at 100°C) for 5 min and MBP surface binding was found in both cases (green). Scale bar, 50 µm. (B) Neurodegeneration after 24-h incubation with 50 µg/mL native or heat-inactivated MBP. Scale bar, 50 µm. (C) Statistical analysis of PI-positive neurons after 24-h incubation with 50 µg/mL native or heat-inactivated MBP (***P<0.001, one-way ANOVA with Dunnett's post-test, n = 4). Data are mean ± SEM.
(TIF)

Figure S5 **MBP binds to sialic acid on neuronal surface.** (A) CTB specifically binds to GM1 through sialic acid. Pre-incubation of MBP blocked binding of CTB on neuronal surface, suggesting MBP may bind to GM1. Neurons were pre-incubated with 50 µg/mL BSA or MBP for 30 min and treated with 1 µg/mL FITC-conjugated CTB for another 30 min. (B) Pre-incubation of CTB did not significantly block MBP surface binding, indicating other binding partners of MBP may be involved. Neurons were pre-incubated with 5 µg/mL FITC-conjugated CTB for 30 min and treated with 10 µg/mL MBP for 5 min. After wash and fixation, surface staining of MBP was performed. (C) Pre-incubation of CTB did not rescue neurotoxicity induced by MBP. Neurons were pre-treated with 5 µg/mL FITC-conjugated CTB for 30 min and then incubated with 50 µg/mL MBP for 24 h. Neuronal cell death was assessed by DAPI/PI double-staining. (***P<0.001, ns, not significant; two-way ANOVA with Bonferroni's post-test; n = 4). Data are mean ± SEM. (D) Neurons were treated with neuraminidase (0.5 U/mL in HBSS) for 1 h, fixed and subject to GD1a surface immunostaining to confirm the removal of terminal sialic acid by enzyme. GD1a immunostaining on neuronal surface was remarkably decreased, indicating effective cleavage of terminal sialic acid by neuraminidase. (E) Neurons were pre-incubated with neuraminidase (0.5 U/mL in HBSS) for 1 h and treated with 10 µg/mL MBP for 5 min. Neuraminidase treatment significantly reduced surface binding of MBP, suggesting sialic acid may be the main binding partner of MBP on neuronal surface. Scale bar, 50 µm.
(TIF)
