# Supplementary figures and images for: Myelin Basic Protein Induces Neuron-Specific Toxicity by Directly Damaging the Neuronal Plasma Membrane
Source: PLoS One. 2014 Sep 25;9(9):e108646. doi: 10.1371/journal.pone.0108646 (PMC4177931; doi:10.1371/journal.pone.0108646)

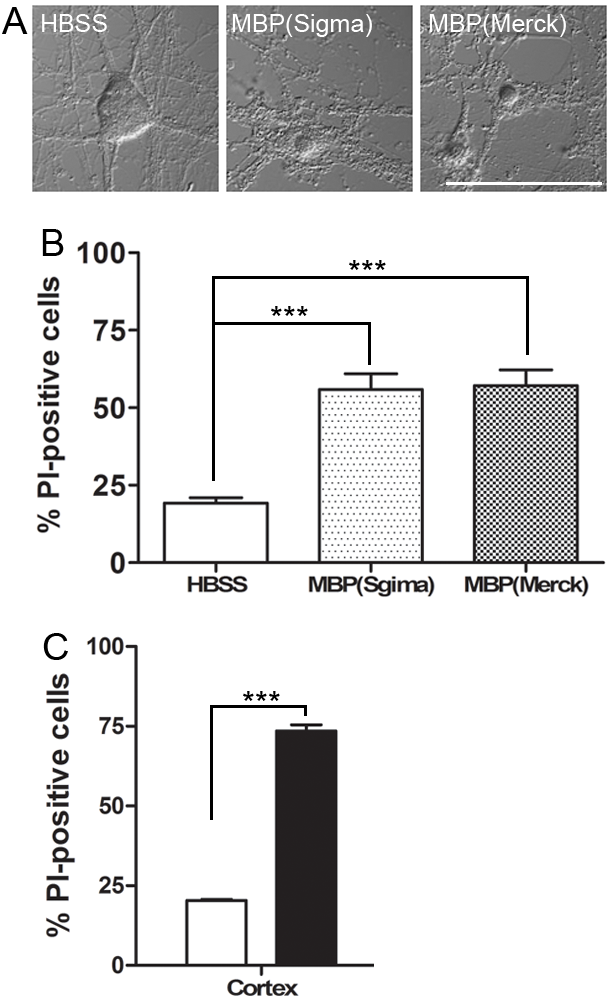

Supplement: Figure S1 — Further confirmation of MBP-induced neurotoxicity. (A) Neurodegeneration of hippocampal neurons after 24-h incubation with 50 µg/mL MBP purchased from either Sigma or Merck. Scale bar, 50 µm. (B) Statistical analysis of PI-positive neurons after 24-h incubation with 50 µg/mL MBP from either Sigma or Merck (***P<0.001, one-way ANOVA with Dunnett's post-test, n = 5). (C) Statistical analysis of PI-positive cortical neurons after 24-h incubation with 50 µg/mL MBP (***P<0.001; unpaired, two-tailed t-test, n = 3). Data are mean ± SEM. (TIF) [file pone.0108646.s001.tif]

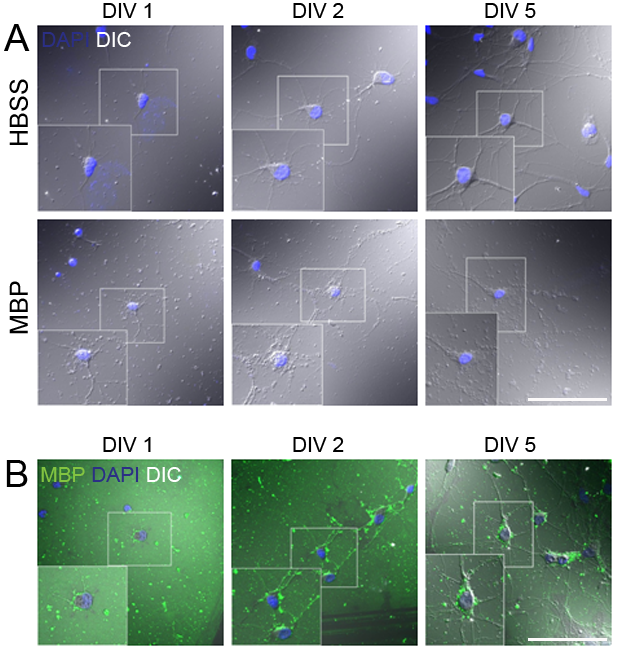

Supplement: Figure S2 — MBP-induced neurotoxicity is independent of neuronal development in vitro . (A) Morphology of hippocampal neurons at DIV 1, 2 and 5 after incubation with 50 µg/mL MBP for 24 h. (B) Surface binding of MBP on neurons at DIV 1, 2 and 5 after incubation with 10 µg/mL MBP for 5 min. Scale bar, 100 µm. (TIF) [file pone.0108646.s002.tif]

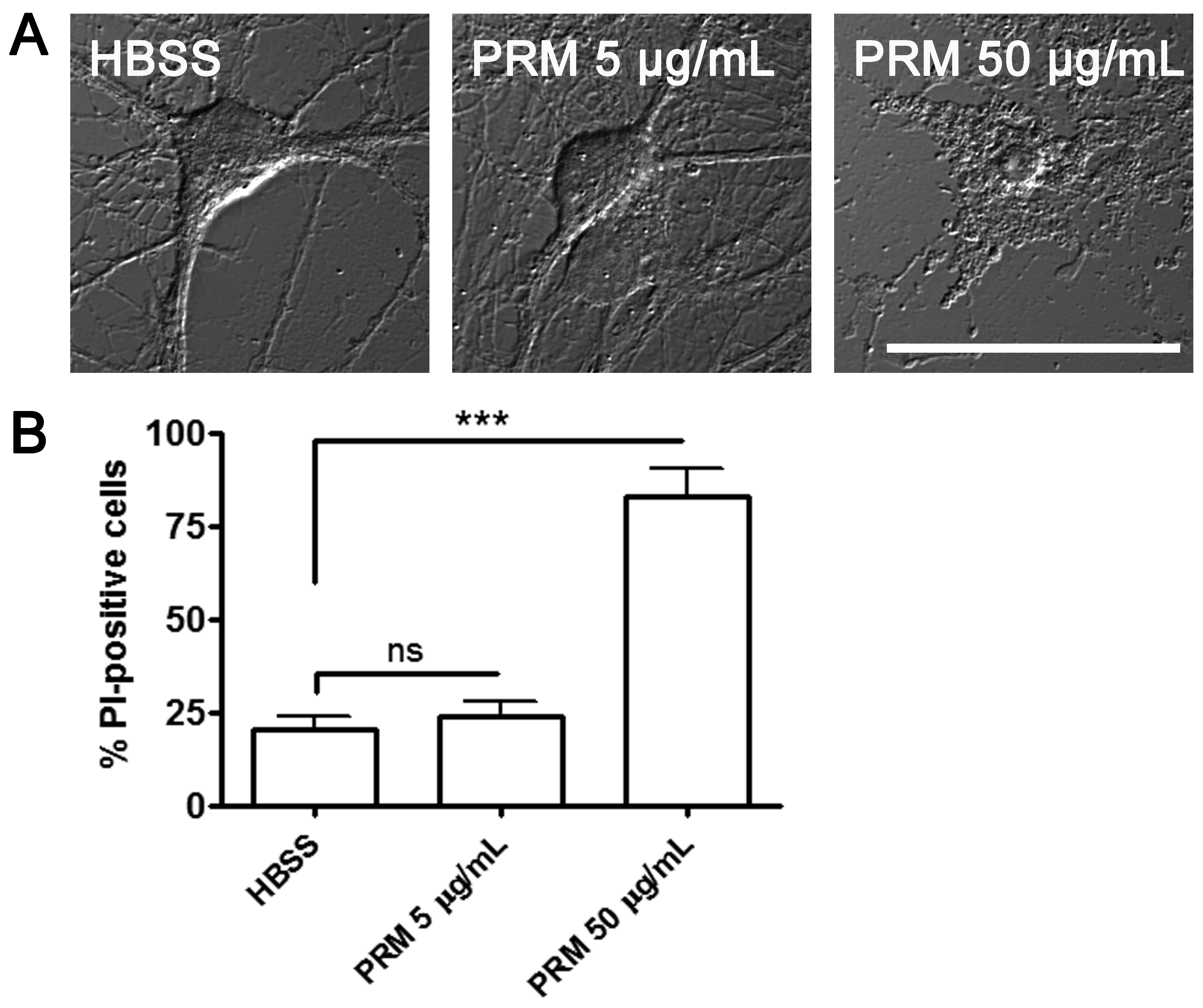

Supplement: Figure S3 — PRM induces neurotoxicity. (A) Neurodegeneration of hippocampal neurons after 24-h incubation with HBSS, 5 µg/mL or 50 µg/mL PRM. Scale bar, 50 µm. (B) Statistical analysis of PI-positive neurons after 24-h incubation with HBSS, 5 µg/mL or 50 µg/mL PRM. (***P<0.001, ns, not significant; one-way ANOVA with Dunnett's post-test, n = 4). Data are mean ± SEM. (TIF) [file pone.0108646.s003.tif]

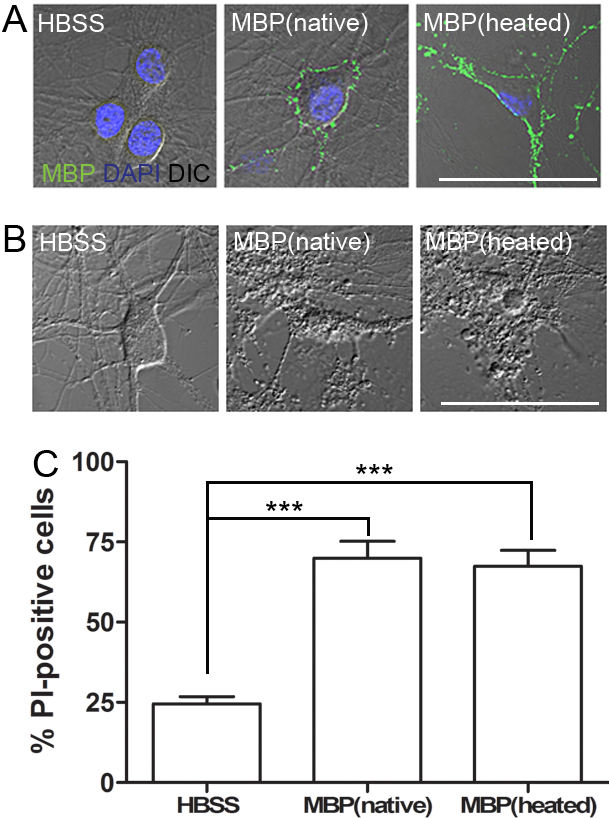

Supplement: Figure S4 — Neurotoxicity induced by heat-inactivated MBP. (A) Hippocampal neurons were incubated with 10 µg/mL native or heat-inactivated MBP (30 min at 100°C) for 5 min and MBP surface binding was found in both cases (green). Scale bar, 50 µm. (B) Neurodegeneration after 24-h incubation with 50 µg/mL native or heat-inactivated MBP. Scale bar, 50 µm. (C) Statistical analysis of PI-positive neurons after 24-h incubation with 50 µg/mL native or heat-inactivated MBP (***P<0.001, one-way ANOVA with Dunnett's post-test, n = 4). Data are mean ± SEM. (TIF) [file pone.0108646.s004.tif]

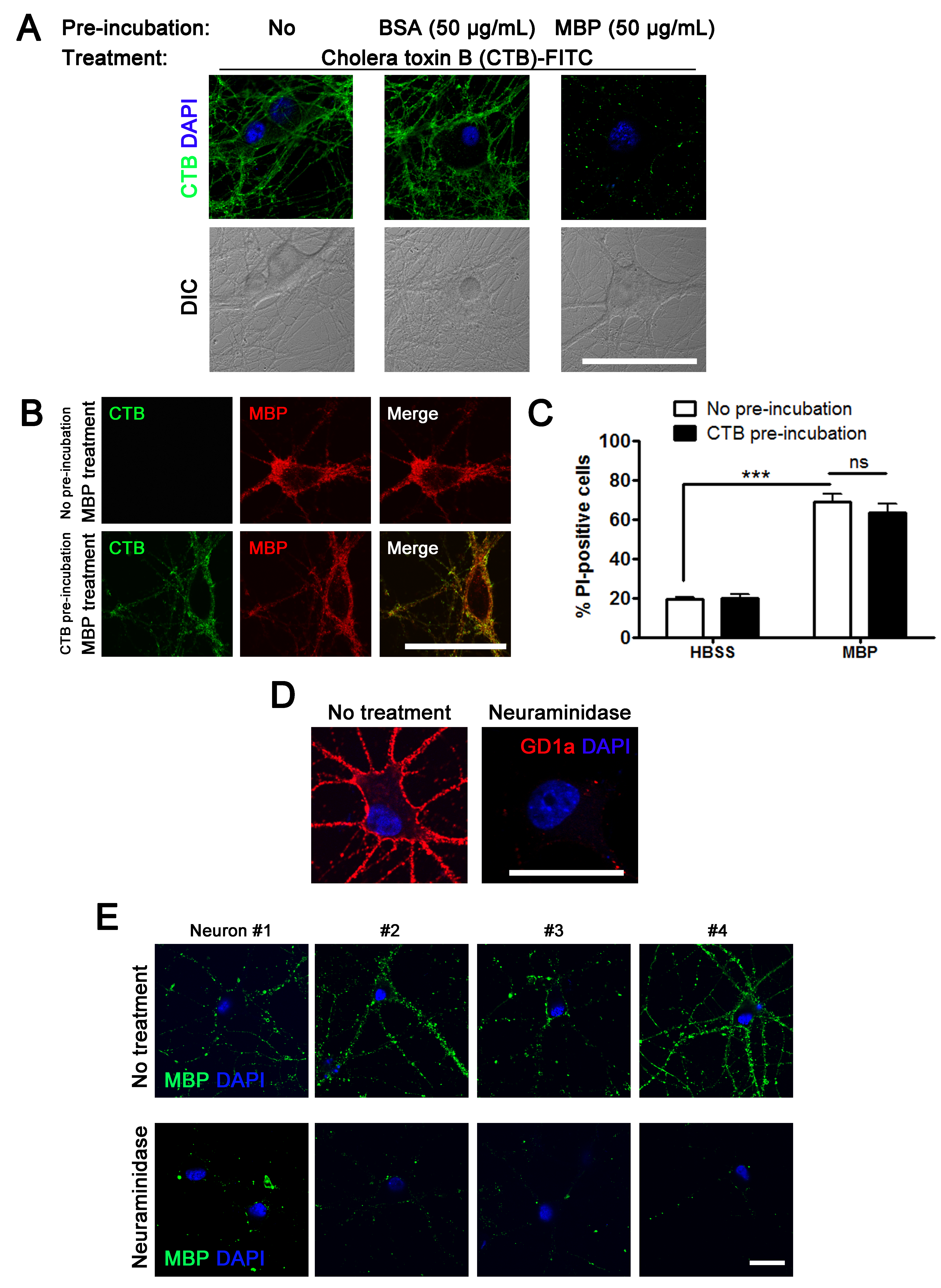

Supplement: Figure S5 — MBP binds to sialic acid on neuronal surface. (A) CTB specifically binds to GM1 through sialic acid. Pre-incubation of MBP blocked binding of CTB on neuronal surface, suggesting MBP may bind to GM1. Neurons were pre-incubated with 50 µg/mL BSA or MBP for 30 min and treated with 1 µg/mL FITC-conjugated CTB for another 30 min. (B) Pre-incubation of CTB did not significantly block MBP surface binding, indicating other binding partners of MBP may be involved. Neurons were pre-incubated with 5 µg/mL FITC-conjugated CTB for 30 min and treated with 10 µg/mL MBP for 5 min. After wash and fixation, surface staining of MBP was performed. (C) Pre-incubation of CTB did not rescue neurotoxicity induced by MBP. Neurons were pre-treated with 5 µg/mL FITC-conjugated CTB for 30 min and then incubated with 50 µg/mL MBP for 24 h. Neuronal cell death was assessed by DAPI/PI double-staining. (***P<0.001, ns, not significant; two-way ANOVA with Bonferroni's post-test; n = 4). Data are mean ± SEM. (D) Neurons were treated with neuraminidase (0.5 U/mL in HBSS) for 1 h, fixed and subject to GD1a surface immunostaining to confirm the removal of terminal sialic acid by enzyme. GD1a immunostaining on neuronal surface was remarkably decreased, indicating effective cleavage of terminal sialic acid by neuraminidase. (E) Neurons were pre-incubated with neuraminidase (0.5 U/mL in HBSS) for 1 h and treated with 10 µg/mL MBP for 5 min. Neuraminidase treatment significantly reduced surface binding of MBP, suggesting sialic acid may be the main binding partner of MBP on neuronal surface. Scale bar, 50 µm. (TIF) [file pone.0108646.s005.tif]
